# Supplementary material for: Repetitive negative thinking mediates the relationship between self-esteem and burnout in an ecological momentary assessment study
Source: Commun Psychol. 2025 Aug 29;3:134. doi: 10.1038/s44271-025-00318-2 (PMC12397341; doi:10.1038/s44271-025-00318-2)
Supplement: Supplementary file 2 — Reporting summary [file 44271_2025_318_MOESM2_ESM.pdf]

nature portfolio

Corresponding author(s): Malin Brueckmann

Last updated by author(s): Aug 13, 2025

Reporting Summary

Nature Portfolio wishes to improve the reproducibility of the work that we publish. This form provides structure for consistency and transparency in reporting. For further information on Nature Portfolio policies, see our Editorial Policies and the Editorial Policy Checklist.

Statistics

For all statistical analyses, confirm that the following items are present in the figure legend, table legend, main text, or Methods section.

n/a	Confirmed	
☐	☒	The exact sample size (*n*) for each experimental group/condition, given as a discrete number and unit of measurement
☐	☒	A statement on whether measurements were taken from distinct samples or whether the same sample was measured repeatedly
☐	☒	The statistical test(s) used AND whether they are one- or two-sided<br>*Only common tests should be described solely by name; describe more complex techniques in the Methods section.*
☐	☒	A description of all covariates tested
☐	☒	A description of any assumptions or corrections, such as tests of normality and adjustment for multiple comparisons
☐	☒	A full description of the statistical parameters including central tendency (e.g. means) or other basic estimates (e.g. regression coefficient) AND variation (e.g. standard deviation) or associated estimates of uncertainty (e.g. confidence intervals)
☐	☒	For null hypothesis testing, the test statistic (e.g. *F*, *t*, *r*) with confidence intervals, effect sizes, degrees of freedom and *P* value noted<br>*Give P values as exact values whenever suitable.*
☒	☐	For Bayesian analysis, information on the choice of priors and Markov chain Monte Carlo settings
☐	☒	For hierarchical and complex designs, identification of the appropriate level for tests and full reporting of outcomes
☒	☐	Estimates of effect sizes (e.g. Cohen's *d*, Pearson's *r*), indicating how they were calculated

Our web collection on statistics for biologists contains articles on many of the points above.

Software and code

Policy information about availability of computer code

Data collection	Ecological momentary assessment was conducted with the movisensXS app (movisens GmbH, Karlsruhe, Germany).
Data analysis	Data pre-processing and all statistical analyses were performed using R (Version 4.4.3).

For manuscripts utilizing custom algorithms or software that are central to the research but not yet described in published literature, software must be made available to editors and reviewers. We strongly encourage code deposition in a community repository (e.g. GitHub). See the Nature Portfolio guidelines for submitting code & software for further information.

Data

Policy information about availability of data

All manuscripts must include a data availability statement. This statement should provide the following information, where applicable:

- Accession codes, unique identifiers, or web links for publicly available datasets
- A description of any restrictions on data availability
- For clinical datasets or third party data, please ensure that the statement adheres to our policy

The data used and/or analysed during the current study are available on the Open Science Framework (DOI: 10.17605/OSF.IO/EXHWD).

Research involving human participants, their data, or biological material

Policy information about studies with human participants or human data. See also policy information about sex, gender (identity/presentation), and sexual orientation and race, ethnicity and racism.

Reporting on sex and gender	Demographic information was assessed within the baseline assessment. Participants were asked with what gender they identify (“What gender do you identify with?”; response options: “female”, “male”, “diverse”, and “prefer not to respond”).
Reporting on race, ethnicity, or other socially relevant groupings	We did not collect data on race or ethnicity.
Population characteristics	A total of 96 individuals participated in the study (78 female, 14 male, 4 diverse or preferred not to disclose their gender), with a mean age of 22.4 years (SD = 2.8, range = 18–30 years).
Recruitment	Participants were recruited online via social media, email distribution lists from various German universities, and the study participation management portal of the Department of Psychology at Bielefeld University. It can be assumed that mainly people who are generally interested in such studies took part. The results may therefore not be generalizable to the entire population.
Ethics oversight	The study was approved by the Ethics Committee of Bielefeld University (reference number 2022-280).

Note that full information on the approval of the study protocol must also be provided in the manuscript.

Field-specific reporting

Please select the one below that is the best fit for your research. If you are not sure, read the appropriate sections before making your selection.

☐ Life sciences ☒ Behavioural & social sciences ☐ Ecological, evolutionary & environmental sciences

For a reference copy of the document with all sections, see nature.com/documents/nr-reporting-summary-flat.pdf

Behavioural & social sciences study design

All studies must disclose on these points even when the disclosure is negative.

Study description	Ecological momentary assessment (quantitative intensive longitudinal data)
Research sample	This sample consists of university students that took part in an university examination period during the data collection. A total of 96 individuals participated in the study (78 female, 14 male, 4 diverse or preferred not to disclose their gender), with a mean age of 22.4 years (SD = 2.8, range = 18–30 years). The sample is not representative.
Sampling strategy	We used a convenience sample. The sample size was chosen because it offers sufficient statistical power to carry out the planned analyses (specifically under consideration of the amount of data points per individual) and also corresponds to the sample size in comparable studies.
Data collection	This data collection was carried out on the participants' smartphones via the movisensXS app (movisens GmbH, Karlsruhe, Germany).
Timing	Data collection took place during university examination periods between January 2023 and February 2024.
Data exclusions	No data or participants were excluded.
Non-participation	No participants dropped out. Participants completed 6,643 out of 8,064 possible surveys, yielding a response rate of 82.4%.
Randomization	Participants were not allocated into experimental groups.

Reporting for specific materials, systems and methods

We require information from authors about some types of materials, experimental systems and methods used in many studies. Here, indicate whether each material, system or method listed is relevant to your study. If you are not sure if a list item applies to your research, read the appropriate section before selecting a response.

Materials & experimental systems

n/a	Involved in the study
☒	☐ Antibodies
☒	☐ Eukaryotic cell lines
☒	☐ Palaeontology and archaeology
☒	☐ Animals and other organisms
☒	☐ Clinical data
☒	☐ Dual use research of concern
☒	☐ Plants

Methods

n/a	Involved in the study
☒	☐ ChIP-seq
☒	☐ Flow cytometry
☒	☐ MRI-based neuroimaging

Plants

Seed stocks	n/a
Novel plant genotypes	n/a
Authentication	n/a
